# Prescription and clinical impact of chest radiographs in 104 French ICUs: the RadioDay Study

**DOI:** 10.1186/cc9557

**Published:** 2011-03-11

**Authors:** MS Serveaux-Delous, K Lakhal, X Capdevila, JY Lefrant, S Jaber

**Affiliations:** 1 CHU Lapeyronie, Montpellier, France; 2 CHU Caremeau, Nimes, France; 3 CHU Saint Eloi, Montpellier, France

## Introduction

Prescribing daily routine chest radiographs (CXRs) in ICU patients is a matter for debate. We aimed at describing current strategies of CXR prescriptions and their diagnostic and therapeutic impacts in a large panel of French ICUs.

## Methods

We performed a postal survey recording ICU habits of CXR prescription and a snapshot single-day (called RadioDay) observational study analyzing all of the prescribed CXRs.

## Results

### Survey

(*n *= 104 ICUs) CXR prescription was made on a daily routine basis for every patient and only in mechanically ventilated patients in 7% and 37% of the 104 ICUs, respectively. Depending on the ICUs, ICU admission (55% of the ICUs), endotracheal intubation (87%), tracheostomy (87%), superior vena cava device (96%), nasogastric tube (48%), chest tube insertion (98%) and chest tube removal (60%) were systematically followed by a CXR. A written procedure for CXR prescription was available in 12% ICUs.

### Snapshot study

On RadioDay, 854 CXRs were performed (8.2 ± 4.6 per center) in 804 patients: 36.5% were prescribed on a daily routine basis. The most frequent indications for on-demand CXR were: follow-up of pleuropulmonary pathology (32%), control after invasive device placement (20%), search for an etiology of respiratory or circulatory failure (13%) and ICU admission (11%). On-demand CXRs were mostly (62%) performed during the morning round. On-demand CXR showed more frequently a tissue abnormality than daily routine CXR (69 vs. 48%, *P *< 0.001) and this radiographic finding was unexpected in 20 and 15%, respectively (*P *= 0.22). On-demand CXR was more frequently associated with treatment modification (which would not have occurred without the CXR) than daily routine CXR (38 vs. 19%, *P *< 0.001): placement/modification/or removal of an invasive device (18 vs. 9%, *P *< 0.001), prescription of another paraclinical investigation (15 vs. 3%, *P *< 0.001), initiation/continuation/or discontinuation of medications (25 vs. 11%, *P *< 0.001). CXR findings were expected and had no impact on management in 56 and 77% (*P *< 0.001) of the on-demand and daily routine CXR, respectively.

## Conclusions

There is an obvious lack of consensus for CXR prescription in French ICUs. The clinical impact of on-demand CXR is higher than that associated with a daily routine prescription.

